# EEG microstates associated with intra- and inter-subject alpha variability

**DOI:** 10.1038/s41598-020-58787-w

**Published:** 2020-02-12

**Authors:** Pierpaolo Croce, Angelica Quercia, Sergio Costa, Filippo Zappasodi

**Affiliations:** 1 Department of Neuroscience, Imaging and Clinical Sciences,“G. d’Annunzio” University of Chieti-Pescara, Chieti-Pescara, Italy; 2 Institute for Advanced Biomedical Technologies, “G. d’Annunzio” University of Chieti-Pescara, Chieti-Pescara, Italy

**Keywords:** Neuroscience, Biomedical engineering

## Abstract

Variation of the magnitude of posterior alpha rhythm (8–12 Hz) has functional and behavioural effects in sensory processing and cognitive performances. Electrical brain activity, as revealed by electroencephalography (EEG), can be represented by a sequence of microstates of about 40–120 ms duration, in which distributed neural pools are synchronously active and generate stable spatial potential topographies on the scalp. Microstate dynamics may reflect transitions between global states characterized by selective inhibition of specific intra-cortical regions, mediated by alpha activity. We investigated the intra-subject and inter-subject relationship between microstate features and alpha band. High-density EEG signals were acquired in 29 healthy subjects during ten minutes of eyes closed rest. Individual EEG signal epochs were classified into four groups depending on the amount of occipital alpha power, and microstate metrics (duration, coverage and frequency of occurrence) were calculated and compared across groups. Correlations between alpha power and microstate metrics between individuals were also performed. To assess if microstate parameter variations are specific for the alpha band, the same analysis was also performed for theta and beta bands, as well as for global field power. We observed an increase in the metrics of microstate, previously associated to the visual system, with the level of intra-subject amplitude alpha oscillations, together with lower coverage of microstate associated with executive attention network and a higher frequency of microstate associated with task negative network. Other modulation effects of broad-band EEG power level on microstate metrics were observed. These effects are not specific for the alpha band, since they can equally be attributed to fluctuations in other frequency bands. We can interpret our results as a regulation mechanism mediated by posterior alpha level, dynamically interacting with other frequency bands, responsible for the switching between active areas.

## Introduction

The dynamics of human brain signals uncover organized fluctuations both at rest and during task or sensory input. Such time signal variability has been recently linked to the efficiency of functional abilities and directly associated to cognitive performances in functional Magnetic Resonance Imaging (fMRI), Magnetoencephalographic (MEG), Electrocorticographic (ECoG) and Electroencephalographic (EEG) studies (for a review see Buzsáki and colleagues^1^).

Alpha rhythm (8–12 Hz) is a prominent component of the spectral content of electrical brain signals, as recorded at the scalp surface by EEG or MEG^2^. Although the parieto-occipital alpha rhythm has been supposed for years to reflect an idle status, it is currently considered to play a crucial role in higher cognitive processes and memory performances and to have a functional significance even at rest^3,4^. Specifically, perception and other ongoing neural activities have been related to alpha rhythm modulation^5^. In this regards, Hanslmayr^6^ hypothesised that states of decreased pre-stimulus alpha oscillations indicate enhanced neural excitability, resulting in improved perceptual acuity. Instead, Jensen and co-workers^7^ proposed that the increase of alpha power over task-irrelevant areas can be interpreted as a mechanism of active inhibition. Indeed, in task with externally-oriented attention, alpha power is reduced over task-irrelevant areas^8–12^. As for memory function, an increase in alpha band activity has been observed in working memory maintenance tasks^13^.

Investigation of alpha rhythm revealed both an intra-subject and an inter-subject’s variability so that a person’s alpha feature cannot be considered as an unchangeable trait like fingerprints^5,14^. Indeed, by looking at EEG recordings at rest, it is evident that different time intervals give rise to a certain degree of alpha variability, even within the state of a closed eyes relaxed vigilance. This variability is probably due to different psychological and physiological states at rest during the recording related to alpha-band modulation, possibly originating from the underlying inhibition mechanisms. Moreover, inter-subject’s alpha variability has been related to both genetics and cognitive measure, e.g. working memory performance^15^.

The brain dynamic as detected by EEG can be globally described as a sequence of “microstates”^16,17^. In microstate analysis, the EEG activity is segmented into periods of approximately 40–100 ms duration, in which distributed neural pools are synchronously active and generate fixed spatial potential topographies on the scalp. Therefore, a single microstate, corresponding to a specific EEG topographical map, may be associated with a “quasi-stable” functional state, in which the brain enters during a specific neural process^18^. As a result, the ongoing EEG time course can be represented by a non-casual sequence of such topographies without any type of a priori hypothesis^19^, for example a priori choice of electrodes of interest, as well as specific time intervals or frequency bands. In the healthy adult resting state, most of the variance of EEG signals has been demonstrated to be explained by sequences of only four specific topographies with fixed polarities, labelled as A, B, C, D^17^. Microstate metrics can be calculated as global descriptors of their dynamics, as mean duration, mean number of microstates per second (occurrence) and coverage^20^. The duration parameter is defined as the mean of duration that a microstate remains stable whenever it appears during the recordings^18^. As such, the microstate duration metric reflects the stability of the activity of the neural assemblies underlying that microstate. The occurrence is calculated as the average number of times per second that a given microstate is dominant and may reflect the tendency of its underlying neural generators to be activated^16^. Coverage is calculated as the percentage of total recording time that a microstate is dominant during a certain condition^18^ That is, it indicates the relative presence (i.e. the relative time coverage) of a microstate with respect to the others.

Recently, a correlation between the four microstate topographies and the blood oxygenated level dependent (BOLD) activations in regions belonging to different human resting state brain networks has been found^21,22^. Indeed, modularity of brain dynamics in behavioural and cognitive control has been evidenced through microstate analysis and microstates have been proved to be useful in the study of brain diseases^20,23–28^.

Recent literature^25^ described intra-subject variability of microstate dynamics within different brain functional status, as found in sleep stage^29^, due to different modalities of task processing, i.e. object-visualization, spatial-visualization, verbalization, rest^30^. Even cognitive manipulation affects microstate dynamics. In particular, modulation of the visual sensory input by the opening of the eyes induced a decrease of explained variance and increment of the metrics associated with microstate B^25^. Finally, distinct pre-stimulus microstates dissociated correct identification of stimuli with and without awareness^31,32^.

**Figure 1:**
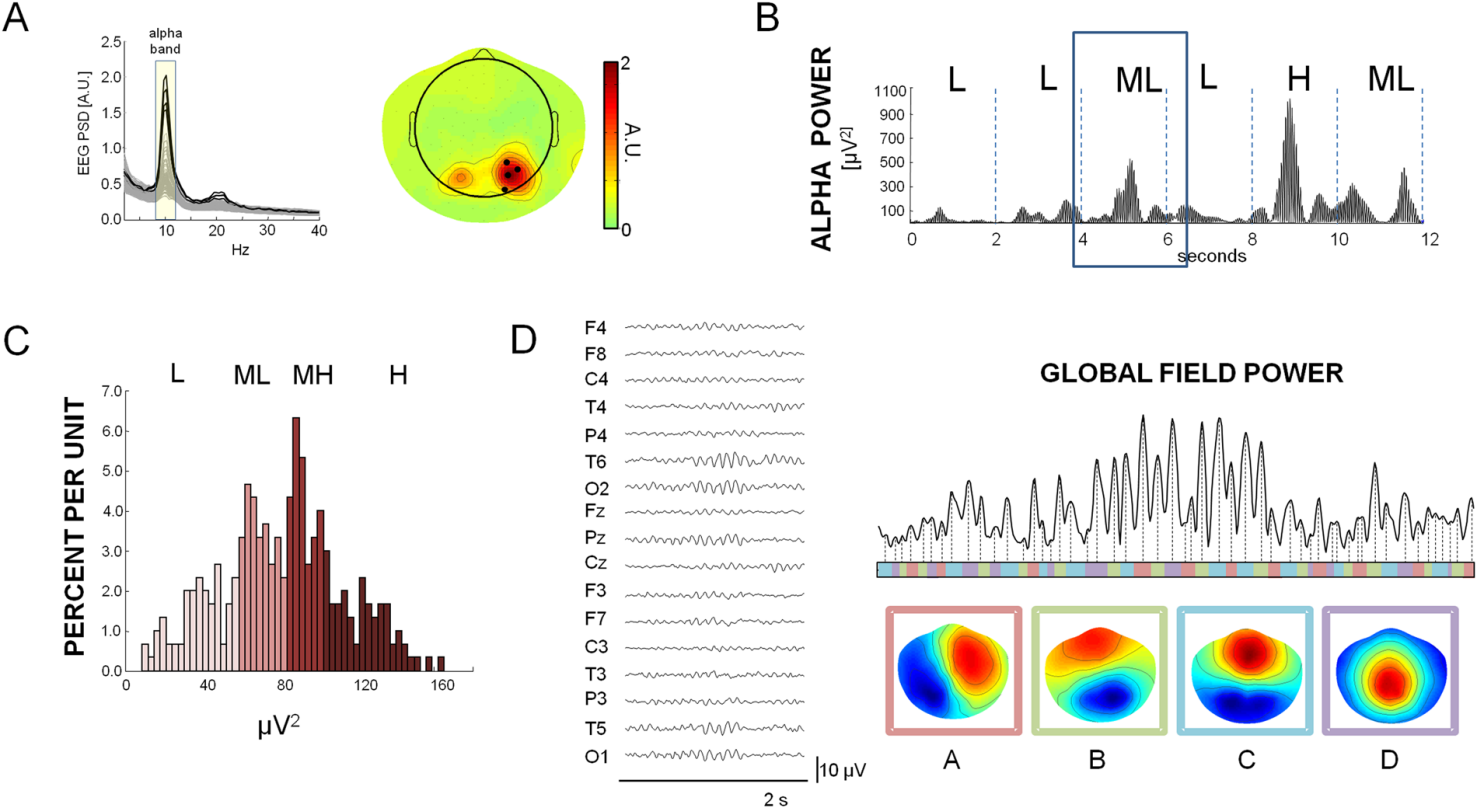
(**A**) For a representative subject (female, 28 years old), Power Spectral Density of the entire EEG recording period (all EEG channels are shown). The four channels with the highest alpha power (represented in bold) were identified. Right: Topographical distribution of the EEG power in the alpha band (8–12 Hz) for the representative subject. The 4 black circles indicate the EEG channels of maximal alpha power selected for EEG segment classification. (**B**) For the representative subject, a 12 second time course of the mean alpha power of the 4 channels shown in A is displayed. Alpha powers in windows of 2 seconds are calculated to obtain an individual empirical distribution. (**C**) For the representative subject, an empirical histogram representing the empirical distribution of the alpha power is shown. The 2-second windows of EEG signals are classified on the basis of the alpha individual distribution as L (low alpha: 0–25 percentile of distribution), ML (medium-low alpha: 25–50 percentile), MH (medium-high alpha: 50–75 percentile), H (high alpha: 75–100 percentile). (**D**) Example of microstate analysis in the 2-second window in the box of panel B is shown. On the left the time course of 17 representative EEG channels over 128 channels, average referenced, is dysplayed. The Global Field Power was computed for each time point (Right top) as standard deviation of the EEG signal across all the electrodes. Each area of GFP corresponds to an EEG topography which remains stable for that period, indicated with the corresponding colour-code. We report the 4 topographies found for this subject by means of the clustering algorithm (**A**–**D**). Every time point is assigned to one microstate and the EEG time course is thus modelled as a sequence of the 4 found topographies. For each microstate the analysis calculates three metrics (the duration, the number of times it occurred per second, the percentage of total time that it is covered by the state), as well as the global variance of EEG data explained by the microstate.

A very recent study^33^ showed that intra-cortical alpha oscillations principally explain the occurrence of different microstates. Since alpha likely reflects decreased cortical excitability, authors suggested that each microstate selectively reflects the inhibition of specific intra-cortical regions. Moreover, they have speculated that microstate sequences, reflecting transitions among four global attractor states, may reflect the dynamics of deactivation or inhibition in default mode network hubs^33^. In this scenario, we posed the hypothesis that microstate dynamics can be related to the amount of posterior alpha activity. We investigated the intra-subject and inter-subject relationship between microstate features and alpha band. In particular, we wanted to clarify whether the microstate features are different in periods of eyes closed rest EEG with different amount of alpha rhythm within healthy subjects and whether a relationship between alpha power and microstate features can be found across subjects. With this aim, high-density EEG (128 channels) was acquired in 29 healthy subjects during ten minutes of eyes closed rest. We compared canonical metrics extracted from microstate analysis across different amounts of alpha power. First, alpha band power was obtained at the individual level by the 4 EEG channels with maximal alpha power (Fig. 1A). The ensemble of alpha power values of all the 2 second epochs were used for each subject to obtain an empirical alpha power distribution (Fig. 1B,C). We classified individual 2 second EEG signal epochs into four groups depending on the 4 quartiles of the alpha power distribution (low, L; medium-low, ML; medium-high, MH; high, H), obtained at the individual level (Fig. 1B,C). Next, conventional microstate analysis was separately performed for each condition (L, ML, MH, H) and four canonical topographies were found for each of the 4 groups, resulting in 16 microstates for each individual (Fig. 1D). Then, microstate metrics (duration, coverage and frequency of occurrence) were calculated and compared across conditions, testing for significant correlation between microstates and the alpha power within individuals. Finally, the correlation between alpha power and microstate metrics across individuals was also performed. Since phase-amplitude coupling between different bands has been evidenced in EEG and MEG signals^22,34^ and may result in oscillatory hierarchy of brain oscillations, a relationship between microstate metrics and alpha levels could be not specific for the alpha band and could be found also for other bands. Indeed, power analyses in the different frequency bands are done in the time domain separately for a given electrode. On the contrary, microstate analysis is done in the spatial domain, giving a global description of EEG signals. Therefore, every frequency band contributes to and is represented in microstates. Therefore, to clarify how specific the results are for the alpha band, the analysis was repeated also for theta (4–7.5 Hz) and beta (13–25 Hz) bands, as well as for the global field power levels.

## Results

Microstate analysis was separately done in 2-second EEG epochs, classified dependently on alpha power amount (L: low, ML: medium-low, MH: medium-high, H: high, Fig. 1).

### Number of microstates

**Figure 2:**
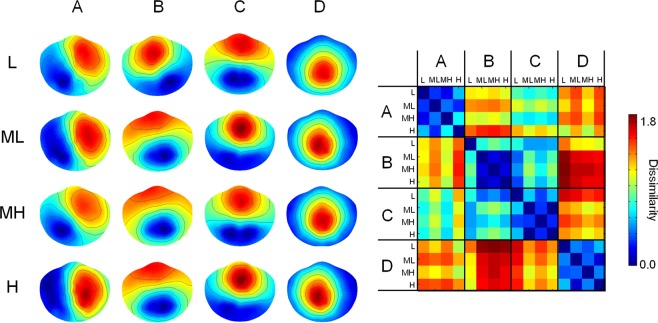
The mean of the topography of the 4 microstates (**A**–**D**) obtained for each alpha level (L, ML, MH, H). On the right, the dissimilarity matrix between couples of topographies is shown. The dissimilarity index between 2 maps ranges between 0 (equal maps) to 2 (maximal dissimilar maps, i.e. opposite maps).

The EEG variance explained by microstates (Global Explained Variance, GEV) increases as a function of the number of templates between 1 and 12. The GEV increment for every addition of 1 template is below the 3.5% for templates 4 to 6 and stays below 1% for templates 6 to 12. According to the KL criterion, the optimal number of templates was found to be equal to 4 for each alpha level (L, ML, MH, H). The 4 templates had topographies similar to those obtained in previous works^16,18^: template A had a left occipital-parietal (negative) to right fronto-central (positive) orientation; template B had the opposite, right parieto-occipital to left fronto-central; template C had symmetrical back to prefrontal orientation; template D a central positivity, with an occipital to fronto-central symmetrical orientation (Fig. 2, left).

### Differences in microstate metrics depending on alpha level

Comparing separately the topographies of each microstate by means of TANOVA, no differences were found for different alpha levels (p > 0.500 for A, B, C and D, consistently). Moreover, the dissimilarity among the topographies within each microstate for different alpha levels was lower than the dissimilarity across different microstates (Fig. 2, right).

The results from the ANOVA designs and post-hoc analyses are described in the following sections for each metric.

#### Duration

**Figure 3:**
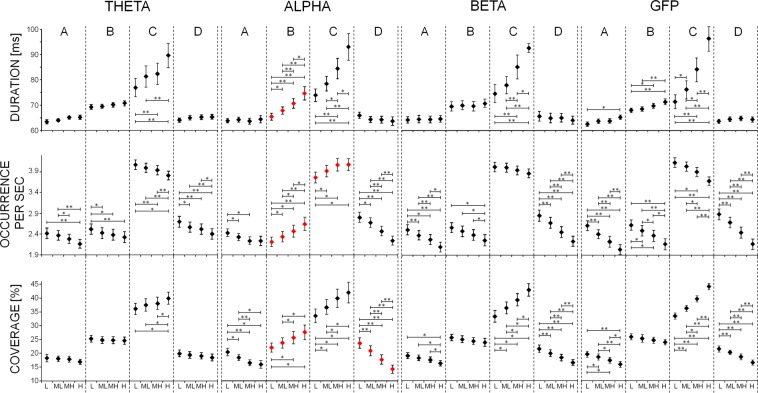
The mean and standard error of microstate metrics in the four conditions of different power (L, ML, MH, H) in different bands (theta, alpha, beta) and global field power (GFP). Stars indicate p-values of paired t-test below the threshold for significance (Bonferroni corrected, *p < 0.05, **p < 0.005). Microstate modulation with alpha power significantly different from other bands and GFP is displayed in red.

A significant interaction *Microstate Class* X *Alpha Level* was found [F (2.0, 54.6) = 9.363; p < 0.001]. Reduced models separately applied for each microstate with *Alpha Level* as a within-subject factor showed a strong *Alpha Level* effect for microstate B [F (1.3, 36.4) = 17.492; p < 0.001] and C [F (1.4, 39.3) = 14.083; p < 0.001]. In particular, as alpha level increased, the duration of microstate B and C increased by about 14% and 26% respectively (Fig. 3). Notably, no effect was found for microstates A and D.

A control analysis was performed to clarify how specific the results are for the alpha band and whether the relationships between microstate durations and alpha level could be found also for other bands. For this reason, the classification procedure of 2-second EEG epochs in 4 different groups on the basis of band power was repeated also for theta (4–7.5 Hz) and beta (13–25 Hz) bands over the 4 channels of maximal alpha power, and microstate metrics were evaluated. Moreover, the higher signal power in the whole frequency range could also account for the differences in microstate metrics. Therefore, the global field power (GFP) was calculated over the 4 channels of maximal alpha and the classification procedure of 2-second EEG epochs in 4 different groups on the basis of GFP was repeated to further delineate whether the microstate metrics are indeed specific for the alpha band or whether they can be merely attributed to an overall increase in signal strength.

For each microstate in which a significant dependence on alpha power level was found, a repeated measure ANOVA was performed with *Band* (Theta, Alpha, Beta, GFP) and *Power Level* (L, ML, MH, H) as within-subject factors. A significant *Band* X *Power Level* interaction evidenced that the modulation of the microstate metric with the power level was different across the 3 bands and GFP. For microstate B duration a significant *Band* X *Power Level* interaction was found [F (1.9, 55.0) = 7.253; p = 0.002]. Post-hoc analysis revealed a specificity of the relationship between microstate B duration and alpha band power, since for the other bands (theta and beta) no differences among different power levels were found (Fig. 3). An increase in microstate B duration was found for different levels of GFP (Fig. 3); however, the increase observed in alpha band was significantly higher than the increase observed in GFP [mean difference and standard error between H and L: 9.2 ± 2.0 ms for alpha band, 3.4 ± 4.6 ms for GFP; sampled t-test t(28) = 2.517; p = 0.018, Fig. 3].

On the contrary, for microstate C a power level increase associated with duration increase was found for all the bands and for GFP (Fig. 3; *Band* X *Power Level* interaction was not significant: p = 0.118).

#### Occurrence

Also for occurrence, a significant interaction *Microstate Class* X *Alpha Level* was evidenced [F (3.8, 106.2) = 18.528; p < 0.001]. Indeed, a strong *Alpha Level* effect was found for microstate B [F (1.4, 40.2) = 15.038; p < 0.001], C [F (1.6, 44.7) = 8.980; p = 0.001] and D [F (1.4, 39.0) = 41.603; p < 0.001], and at less extent A [F (1.6, 44.2) = 5.561; p = 0.012]. Post-hoc comparisons (Fig. 3) showed that microstate C occurrence was low in periods with a low level of alpha. Indeed, for this microstate, the L occurrence values were significantly lower than MH and H values. Moreover, an increase of occurrence for microstate B of about 24% from L to H was found (Fig. 3). The behaviour of microstate B and C occurrence was specific to the alpha band: looking at the theta and beta bands and at GFP, both microstate B and C occurrences of theta band, beta band and GFP showed opposite trends with power level than microstate B and C occurrences of alpha band did [interaction *Band* X *Power Level*: F (2.3, 63.9) = 21.629; p < 0.001 for microstate B and F (1.8, 51.4) = 57.532; p < 0.001 for microstate C]. Microstate C occurrence for beta band did not show significant variation with power level (Fig. 3).

#### Coverage

A significant interaction *Microstate Class* X *Alpha Level* [F (2.7, 76.6) = 16.040; p < 0.001] was found for percentage coverage. Reduced models showed that all the microstate values of coverage changed with the alpha level [microstate A: F (1.4, 40.5) = 11.344; p < 0.001; microstate B: F (1.3, 35.9) = 23.001; p = 0.002; microstate C: F (1.3, 37.2) = 11.485; p = 0.001; F (1.4, 38.8) = 47.265; p < 0.001]. Post-hoc comparison showed that as alpha level increased, microstates B and C were more represented and, on the contrary, microstates A and D were less represented. Indeed, the coverage increase of about 5% and 8% from L to H for classes B and C respectively, while it decreased of about 4% and 9% for classes A and D (Fig. 3). The increase of microstate B coverage with power level was specific to alpha band (interaction *Band* X *Power Level* F (1.9, 52.3) = 25.436; p < 0.001, Fig. 3). Differences across bands were observed also for microstate D coverage reduction [F (2.5, 68.9) = 2.933; p = 0.049]. Indeed, the reduction of microstate D coverage between L and H for alpha band was of about 10%, significantly different from the H vs L reduction observed both in beta band, of about 5% (p < 0.001), in theta band, of about 2% (p < 0.001), and in GFP, of about 5% (p = 0.003).

#### Probability transition and predominance

No difference either in probability transition or in predominance were found for different alpha levels.

### Relationship between alpha power and microstate features across subjects

**Figure 4:**
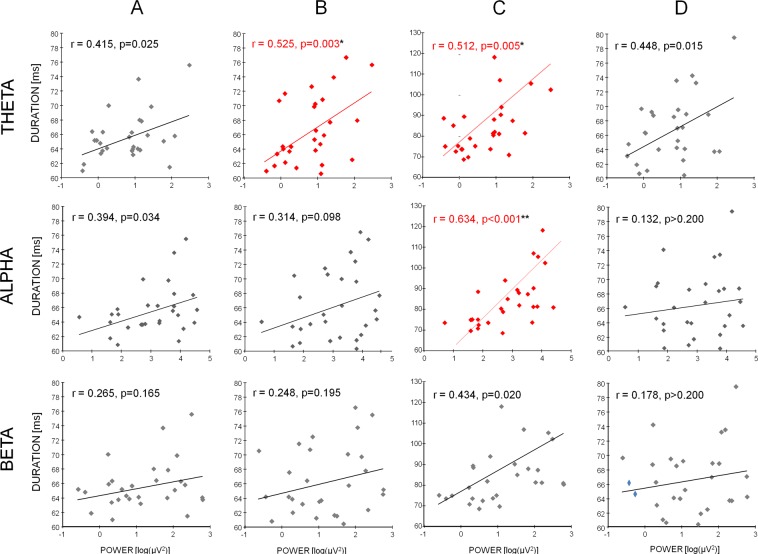
Scatterplot of microstate duration of each subject over individual mean band power and regression lines are shown. Pearson’s r values and uncorrected p are also shown. Significant correlations after Bonferroni correction are displayed in red (*p < 0.05, p < 0.005).

A positive correlation was found between duration of microstates C and alpha power across subjects (Pearson’s r = 0.634, p < 0.001; 95% confidence limit: 0.481, 0.782; Fig. 4). A positive correlation was found also between theta power and microstate C duration (r = 0.512, p = 0.005, Fig. 4). However, since inter-subject alpha and theta powers are strongly correlated (r = 0.800, p < 0.001), in order to control if this relationship is mediated by inter-subject alpha power, the partial correlation between alpha power and microstate C duration, controlled for theta power, was calculated and also the contrary was calculated (i.e. the partial correlation between theta power and microstate C duration, controlled for alpha power). A significant positive correlation was found in the first case (r = 0.442, p = 0.018), but not in the latter (r = 0.094, p = 0.634). Moreover, while no significant correlations were found between alpha power and microstate A, B and D durations (Fig. 4), a positive correlation was found between theta power and microstate B duration (r = 0.525, p = 0.003; 95% confidence limit: 0.481, 0.782; Fig. 4).

No correlations were found between beta power and microstate durations. Also, no correlations were found between band powers and occurrence, coverage and transition times.

## Discussion

In this study, we aimed to characterize microstate metrics in relation to the posterior intrinsic alpha band variability in eyes closed rest condition. Our results showed that, looking at intra-individual alpha variability, the same modulations are observed across different frequency bands for some microstate metrics, but some others seem to change differently with alpha band with respect to other bands. In particular, we observed an increase in the metrics of microstate B, a decrease of coverage of microstate D and an increase of frequency of microstate C with the level of intra-subject amplitude alpha oscillations. Other microstate metrics (microstate C duration and coverage, microstate A and D frequency, microstate A coverage) are not specific for the alpha band, since they can equally be attributed to fluctuations in other frequency bands and modulation effects of broad-band EEG power level on microstate metrics can be observed. Even if the alpha band is the most prominent component in the power spectrum of EEG at eyes closed, the other frequency bands likewise contribute to the overall variation of the EEG signal and modulate the microstate metrics. Our data showed that microstates and frequency bands are independent measures of the EEG signals at rest that cannot be related to one another in a simple and direct fashion. Specific studies and methods addressing this point are needed^34^.

We found that microstate A duration did not change with band power and occurrence and coverage decreased across the frequency bands and the GFP. Occurrence and coverage of microstate B increased with alpha power, and not with theta, beta power or GFP. Microstate B duration also increased with alpha, but also with GFP. However, the alpha power is the 60–80% of the power spectrum of the EEG at eyes closed, so a strong relationship between alpha power and GFP is present. Therefore, since the microstate B variation is observed in exactly the same fashion only for GFP and alpha power, but not for theta and beta bands, it can be argued that the variation observed for GFP is essentially due to alpha. The increment of microstate B metrics suggests an increased stability of microstate B in periods with a strong alpha activity. There is no general agreement on the functional significance of microstate B and on its relationships with resting state networks^21,22,25^. In this regard, Seitzman and colleagues^25^ observed that the switching from an eyes-closed state to an eyes-open state was significantly related to an increase in the microstate B coverage and occurrence, suggesting the association of microstate B with the processing of visual inputs. The idea of an association between microstate B and the visual system is confirmed by the findings of Britz and co-workers^21^, which show a correlation between microstates B with visual area and the visual network identified by Mantini^35^. Nevertheless, in Milz^33^, no increase in microstate B metrics was observed during mental visualization of images previously presented on a screen. It is not univocal in the literature whether a single microstate topography corresponds to the activity of a set of brain regions or to a specific resting state network, as revealed by fMRI, or to an ensemble of them^21,22^. However, it is well established that different topographies of microstate mirror distinct neuronal network activities and thus reflect different cognitive processes or mental states^21,22,33^. Indeed, a general quoted hypothesis, that consider the brain dynamics as the result of interactions of functional networks organized in a hierarchical architecture, is that the microstates are the “building blocks of thought”^18^. According to this hypothesis, it is assumed that the EEG microstates capture mental states of about 100 ms duration^18^. The analysis of the microstate syntax provides a tool to characterize the sequence of these states, and it is a useful tool to describe the temporal dynamics of spontaneous brain activity. Therefore, dynamics of microstates may reflect the dynamic synchronization of functional networks’ subunits^36^.

Specific effects of modulation of BOLD connectivity mediated by alpha power levels have been evidenced^35,37^. Scheeringa and colleagues^37^ studied whether fluctuations of the posterior EEG alpha power are correlated with connectivity within and between the visual network. This leads us to support the hypothesis that an increased alpha level in a brain region is correlated to a decreased connectivity with other regions. As alpha oscillations are strongly observed over posterior scalp regions, it could be supposed that the visual system is involved in such a process^37^. Mantini and colleagues^35^ positively correlated the alpha power fluctuations with the BOLD signal in regions of default mode, dorsal attention and visual networks. Our results support and extend these ideas as we found a modulation effect specific to microstate B dynamics with alpha level.

Looking at inter-subject variability, microstate B duration was not correlated with alpha power, but microstate B is more stable in subjects with higher theta power. Resting state theta power increase has been associated to decrease in vigilance^5^ and in our knowledge there are not studies systematically investigating inter-subject theta variability at rest and its functional correlate. Further studies are needed to highlight the link between inter-subject theta power and microstate B.

We observed that although microstate C duration and coverage increased with power level in all the bands and GFP, microstate C occurrence was higher during periods of higher alpha activity and on the contrary decreased with other bands and GFP. We may interpret these results as a higher tendency of neural generators of microstate C to activate during periods of higher alpha power. Although several pieces of evidence from different studies (e.g.^23,33,38,39^) suggest that participants tend to spend most of the time during resting in microstate C rather than in A and/or B, the functional significance of microstate C is still unclear. A correlation with positive BOLD activations in the posterior part of the anterior cingulate cortex (ACC) as well as bilateral inferior frontal giri and right anterior insula for microstate C has been shown^33^. These areas were identified as RNS6 in Mantini^35^ and are considered part of the saliency-network^40,41^, a network that plays a crucial role in switching control between central-executive function and the default mode. Nevertheless, Seitzman^25^ proposed that microstate C could reflect a component of the default mode network, considered a task-negative network in which a decrease of activity can be observed during the performance of cognitive tasks. The posterior cingulate is considered a hub of the default mode network^42^. Indeed, memory retrieval fMRI studies have reported important functional differentiation within the default mode network^43–45^. Specifically, posterior regions such as angular gyrus and posterior cingulate/precuneus were active during memory retrieval, whereas anterior regions (prefrontal cortex) were inactive^44^. Here, within the intrinsic variability of the alpha band oscillations, we found an increase of microstate C occurrence as the amount of alpha activity increased. Such modulation of microstate C dynamics with the alpha level could be thought to be in line with the idea that, if microstate C is correlated with the default mode network^25^, it is reasonable to expect an increase in the frequency representation of microstate C with a higher alpha activity, i.e. with an idle status. This is also supported by the findings of Seitzman^25^, who observed a decrease of microstate C dynamics during the execution of a mental subtraction task with respect to a resting state condition. A positive correlation between microstate C duration and alpha power was also found focusing on inter-subject alpha variability, confirming more stability of this microstate in subjects with higher posterior alpha power.

Regarding microstate D, metric increase was observed under resting conditions during a serial subtraction task, independently of eyes-open or closed condition^25^. Microstate D was associated to the dorsal attention network (as also supported by the findings of Britz and collegues^21^). However, in Milz^30^, where a behavioural manipulation study was performed, the duration and occurrence of microstate D increased during rest with respect to levels observed during the various tasks (object-visualization, spatial-visualization, verbalization). These findings suggest that microstate D could represent some aspects of attention, focus switching, and reorientation occurring more frequently during rest than during tasks. Here we found a decrease in microstate D coverage with the level of alpha, beta power and GFP. This decrease was significantly higher in alpha band with respect to the decrease we documented in other bands. Our data support an opposite behaviour of microstate D with respect to microstate C. Such opposite behaviour between these two microstates has already been described in a serial subtraction task^25^, suggesting a negative task modulation role for the alpha level which is reflected in the microstate metrics. Similar effects were found in Mantini^35^ in which a positive correlation was found at rest between alpha power increase and the activity in the default and self-referential networks; whereas an alpha power decrease correlates with an increased activity in areas of dorsal attention network. The authors suggest that default and dorsal attention networks may be coupled in terms of alpha power amount. Since microstate C has been correlated with the saliency network as identified in Mantini^35^ and Britz^21^, we can speculate that an increase in alpha level corresponds to a greater capability of switching between control functions and default mode network. Indeed, the long-range functional antagonism between visual areas and thalamic or other cortical regions may be attenuated by an higher level of inhibition.

Many types of rhythms around 10 Hz have been measured and characterized from different cortical areas in healthy humans both at rest and during tasks^3,46^, but we focused on occipital-parietal alpha rhythm in eyes closed rest condition. Indeed, posterior alpha waves in eyes closed condition at rest are the strongest signals detected by EEG. Recently, such oscillations have been related to cognitive performances, playing an active role in network synchronization and communication and inhibition of areas of the cortex not in use^47^. The difference in microstate metrics, that we found in relation to alpha power, could also be explained considering a different number of microstates for each level of alpha power (L, ML, MH, H). Indeed, a difference of about 5% in explained variance has been found between L and H. This means that more than 4 canonical microstates could be considered when alpha power is low, while in periods of high alpha power, when the signal is more stable, a lower number of microstates are sufficient to explain the global variance of data. According to the literature^16,18,23^, we preferred to consider in our analysis only the 4 canonical microstates, explaining more than 80% of the global variance of data, to make uniform the number of microstate across conditions in order to compare their metrics. Notably, in Koenig^23^ normative values of microstates metrics for different ages in a healthy population are given and, in the age range of our group (between 20 and 30 years), comparable values were found.

To sum up, we observed dependence of microstate B metrics with respect to the level of alpha power, together with a lower relative presence of microstate D with respect to other microstates and a higher tendency of microstate C underlying neural generators to become activated. Given the association between resting state networks and microstate topographies, we quantified how the modulation of alpha activity is reflected in microstate metrics. In particular, the complementary behaviour of microstate B and C with respect to microstates D was found. As microstates correspond to co-activation of a set of cerebral areas, we may interpret such behaviour as a regulation mechanism mediated by posterior alpha level responsible for the switching between active areas. However, since a specificity of relationship with alpha power was not found, we could speculate on a more complex mechanism involving also other frequency bands, dynamically interacting with alpha band.

## Methods

### Subjects and EEG recording

Twenty-nine healthy subjects (aged 24.6 ± 5.2, 9 women and 20 men) participated in the study. They were all right-handed, as confirmed by the score of the Edinburgh Manuality test^48^ and were not receiving any pharmacological treatment at the time of recordings. Subjects sat on a comfortable armchair with eyes closed, while ten minutes of EEG activity was acquired at rest. This was the only purpose of the experiment and subjects did not perform other tasks. The experiment was conducted with the understanding and written informed consent of each participant, according to the Code of Ethics of the World Medical Association, and the standards established by the University of Chieti Institutional Review Board and Ethics Committee. The experimental protocol was approved by the Ethics Committee of “G. d’Annunzio” University of Chieti-Pescara.

The EEG activity was recorded by a 128-channel system (Electrical Geodesic). The impedances were kept below 100 kΩ. EEG data were sampled at 250 Hz and collected for off-line processing.

### Data analysis

Data were visually inspected to exclude saturated epochs of EEG signals from further analysis. A semi-automatic procedure, based on Independent Component Analysis^49^, was applied to identify and remove ocular, cardiac and muscular artefacts. Signals were re-referenced to the common average. The Power Spectral Density (PSD) was estimated for each EEG channel by means of the Welch procedure (Hamming windowing, 0.5 Hz frequency resolution, 50% overlap). Alpha band power was obtained for all EEG channels as the PSD average in the 8–12 Hz frequency band. For each subject, the 4 channels with maximal alpha power were selected. These channels laid over the occipital regions for all subjects (Fig. 1A). To assess the alpha power time variability, the raw traces of these 4 EEG channels, after artefact removal, were band-pass filtered between 8–12 Hz (forward-backward Chebyshev filter of type 2) and squared. Then, the time-average of the squared filtered signals in non-overlapping windows of 2 seconds in duration were computed (Fig. 1B). In this way, the ensemble of alpha power values of all the 2 second epochs were used for each subject to obtain an empirical alpha power distribution (Fig. 1C). The 4 quartiles of this distribution were used to classify the 2-second windows based on the amount of alpha power: from 0 to 25 percentile: low alpha (L); from 25 to 50 percentile: medium-low alpha (ML); from 50 to 75 percentile: medium-high alpha (MH); from 75 to 100 percentile: high alpha (H). These 2-second windows, classified on the basis on the alpha level, were considered for microstate analysis.

In general, microstate analysis aims at identifying epochs of stability of the scalp’s electric potential that lasts typically 40–120 ms. Conventional EEG-microstate analysis determined four typical topographies across the healthy population (namely A, B, C and D^17^) that seem to represent the synchronous activity of coordinated neural ensembles. A Butterworth filter of 2nd order was used to forward and back filter the 128 raw EEG signals between 1 and 30 Hz. Data were down-sampled to a sampling rate of 125 Hz. For each subject and for each of the four alpha levels (L, ML, MH, H), microstates were extracted from the 2-second windows of EEG time course by means of a modified version of the k-means clustering algorithm^50^. First, the Global Field Power (GFP) was calculated for each time frame as the standard deviation of EEG signals across electrodes (Fig. 1D). Only the EEG data corresponding to the maxima of GFP were then submitted to the clustering algorithm. Indeed, these peaks correspond to periods of the highest topographic stability, when the probability to observe a transition is more likely^19^. For each subject and for each condition, the k-means algorithm was repeated varying k from 1 to 12. The optimal number for k was chosen by estimating, for each repetition, the Krzanowski-Lai (KL) criterion and by choosing the number of clusters corresponding to the second KL maximum value^19^. Separately for each condition (L, ML, HM, H), the mean maps (across subjects) were calculated. To do this, a number of initial maps equal to optimal k (k = 4) were randomly chosen. Then, all possible combinations of all subject maps were tested to find the set which best spatially correlated with the initially chosen maps. This set was averaged to obtain a new set of maps. The procedure was repeated until the best fit was found and the new assignment did not change anymore. The maps resulting from the previous procedure (four maps for each alpha level) were paired between the 4 conditions (L, ML, MH, H) by ensuring the minimal maps dissimilarity and were labelled as A, B, C, D in accordance to the topographies previously found in literature (see Koenig^20^ for a complete description). In this way, a total of sixteen maps (four maps, labelled A, B, C, and D for each of the four alpha levels L, ML, MH, H) were obtained. The four global maps representing all four conditions were computed by clustering the sixteen maps again by means of the same procedure just described. To compute microstate metrics, the obtained global maps were fitted backward to the original data calculating the maximum correlation between each template and the topography at each time instant corresponding to the maximum value of GFP. Such a procedure allows us to represent the EEG time course in terms of a sequence of microstates and to extrapolate variables of interest (Fig. 1D). For each subject and for each microstate class, the following metrics were calculated^51^:

1. Median microstate duration: the median of the time covered by a single microstate class. Indeed, at the individual level the distributions of microstate durations are not normally distributed for L, ML, MH and H alpha (consistently, Kolmogorov-Smirnov test, p < 0.05, Fig. 5). For this reason, the mean is not a valid parameter to capture the microstate duration and the median of the duration distributions for the different levels of alpha power was considered as the parameter that best describes the duration.

**Figure 5:**
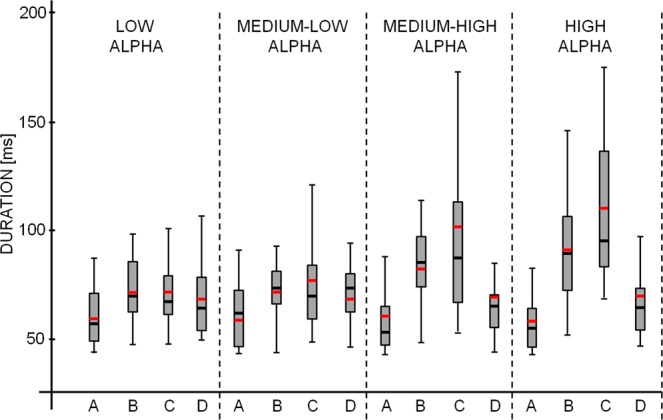
For a representative subject (male, 30 years old), boxplot of microstate durations for L, ML, HL, H alpha power levels are shown. The gray box indicates the 25–75 percentile of distribution and the line the 5–95 percentile. Black horizontal lines show the median value and the red lines the mean. In this example it is evident that, since the duration distribution are not normally distributed (kolmogorv-Smirnov test, p < 0.001 consistently), the mean, instead of the median, generally over-estimates the duration of microstates.
2. Mean occurrence per second: mean number of distinct microstates of a given class occurring within a 2-second window.
3. Mean percentage of covered analysis time: percentage of time covered by a single microstate class.

Mean Global Explained Variance (GEV) was also obtained for all microstate classes, as the sum of the explained variances weighted by the global field power at each moment in time. The metrics were separately computed for each alpha level (L, ML, MH, H).

According to Lehmann^24^ we also evaluated microstate syntax, i.e. the probabilities of transition from one state (represented by a template) to another. Twelve possible transitions were considered (A to B, B to A, A to C, C to A, A to D, D to A, B to C, C to B, B to D, D to B, C to D, D to C). Then the ‘directional predominance’ between two microstates X and Y was evaluated^24^ as the difference between the X to Y and Y to X transition probability. Directional predominance quantifies the directional asymmetries in the transitions between two microstates. Six possible directional predominance values were evaluated, corresponding to the six possible microstate pairs (A ↔ B, A ↔ C, A ↔ D, B ↔ C, B ↔ D, C ↔ D). A significant positive value of X ↔ Y corresponds to a higher probability to transit from X to Y with respect to transit from Y to X, and a negative value corresponds to the opposite predominance to transit from Y to X.

### Statistical analysis

Differences in microstate topographies between conditions were assessed by Topographical Analysis of Variance (TANOVA^52^). Moreover, the difference between two map u and map v was quantified by means of the dissimilarity index, defined as follows:

$${d}_{{\rm{u}},{\rm{v}}}=\,\sqrt{\frac{1}{N}\mathop{\sum }\limits_{i=1}^{N}{(\frac{{u}_{i}}{GF{P}_{u}}-\frac{{v}_{i}}{GF{P}_{v}})}^{2}}$$

where, *u*_*i*_ and *v*_*i*_ are the electric potentials of the *i*_*th*_ electrode for the maps *u* and *v* respectively, *GFP*_*u*_ and *GFP*_*v*_ are the global field powers of the maps and *N* is the number of EEG electrodes. The dissimilarity ranges between 0 (equal maps) to 2 (maximal dissimilar maps, i.e. opposite maps).

To assess differences in microstate metrics in EEG epochs with different levels of alpha power, repeated measure Analyses of Variance (ANOVAs) were separately performed for each microstate metric (duration, occurrence and coverage). A 4 × 4 design was applied, with *Microstate Class* (A, B, C, D) and *Alpha Level* (L, ML, HL, H) as within-subject factors. The Greenhouse-Geisser correction has been applied if the sphericity assumption was not valid. When an interaction *Microstate Class* X *Alpha Level* was found, reduced models were separately applied for each microstate class, with *Alpha Level* as a within-subject factor. Post-hoc paired samples t-tests were carried out to assess significant differences between microstate class metrics among alpha levels. Post-hoc comparisons were Bonferroni corrected. The same ANOVA design was applied to transition probabilities and directional predominance, considering the within-subject factor *Pairs* (12 transition probabilities or 6 microstate pairs) instead of *Microstate Class*.

As control analysis, to assess that the relationships found between power levels and microstate metrics were specific for alpha band, the procedure of classification of 2-second EEG epochs in 4 different groups on the basis of band power was repeated also for theta (4–7.5 Hz) and beta (13–25 Hz) bands over the 4 channels of maximal power. Microstate metrics were evaluated for each of the 4 conditions (L, ML, HL, H) for both theta and beta band. For each microstate in which a significant dependence on alpha power level was previously found, a repeated measure ANOVA was performed with *Band* (Theta, Alpha, Beta) and *Power Level* (L, ML, MH, H) as within-subject factors. A significant interaction *Band* X *Power Level* evidenced that the modulation of the microstate metric with the power level was different across the 3 bands. The Bonferroni post-hoc analysis was then applied.

To assess a relationship between alpha power and microstate features across subjects, the following procedure was applied. First, the mean values of alpha power (i.e. average across the 4 Alpha Levels) and of microstate metrics were found for each subject. Second, after a check of the normal distribution of the values by means of the Kolmogorov-Smirnov test, the Pearson’s correlation across subjects was calculated between each metric and the alpha power values. Bonferroni correction was applied separately for each metric, as duration, occurrence and coverage are not independent measures, which Bonferroni correction assumes. A threshold for significance was assessed at p < 0.013 (i.e. 0.05/4, 4 being the number of microstates). A percentile-based bootstrap, with 5000 replicate samples, was applied to assess the 95% confidence interval of Pearson’s r-values. The procedure was repeated also for theta and beta bands.

## Data Availability

The data are available at request.
